# Biomolecular engineering of drugs loading in Riboflavin-targeted polymeric devices: simulation and experimental

**DOI:** 10.1038/s41598-022-09164-2

**Published:** 2022-03-24

**Authors:** Mohammad Khedri, Mostafa Keshavarz Moraveji

**Affiliations:** Department of Chemical Engineering, Amirkabir University of Technology (Tehran Polytechnic), No. 350, Hafez Ave, Valiasr Square, 15916-34311 Tehran, Iran

**Keywords:** Computational biology and bioinformatics, Molecular medicine, Materials science, Mathematics and computing, Nanoscience and technology

## Abstract

The synthesis of polymeric nanoparticles (NPs) with efficient drug loading content and targeting moieties is an attractive field and remains a challenge in drug delivery systems. Atomistic investigations can provide an in-depth understanding of delivery devices and reduce the number of expensive experiments. In this paper, we studied the self-assembly of poly (lactic-co-glycolic acid)-b-poly (ethylene glycol) with different molecular weights and surface compositions. The innovation of this molecular study is the loading of an antitumor drug (docetaxel) on a targeting ligand (riboflavin). According to this work, a novel, biocompatible and targeted system for cancer treatment has been developed. The obtained results revealed a correlation between polymer molecular weight and the stability of particles. In this line, samples including 20 and 10 w/w% moiety NPs formed from polymers with 3 and 4.5 kDa backbone sizes, respectively, are the stable models with the highest drug loading and entrapment efficiencies. Next, we evaluated NP morphology and found that NPs have a core/shell structure consisting of a hydrophobic core with a shell of poly (ethylene glycol) and riboflavin. Interestingly, morphology assessments confirmed that the targeting moiety located on the surface can improve drug delivery to receptors and cancerous cells. The developed models provided significant insight into the structure and morphology of NPs before the synthesis and further analysis of NPs in biological environments. However, in the best cases of this system, Dynamic Light Scattering (DLS) tests were also taken and the results were consistent with the results obtained from All Atom and Coarse Grained simulations.

## Introduction

In the last few decades, polymeric (micro- and nano) particles have attracted attention in biomedical applications due to their tunable properties^1–3^. They possess a wide range of advantages, such as biocompatibility, biodegradability, controlled release, and targeted particles. Among numerous polymers, poly (lactic-co-glycolic acid) (PLGA) is well known for its properties owing to its constituents: lactic acid (LA) and glycolic acid (GA), which are hydrophilic and hydrophobic parts, respectively^4^. Various drug delivery systems based on this polymer have been approved by the FDA, and others are in (pre)clinical trials to enter the market^2,5–7^.

A large number of experimental analyses have been performed to produce successful NPs for drug delivery applications. However, there is still ambiguity and a lack of information on the interactions of polymer chains with each other (within the structure), with surrounding environments, and finally with the target organ tissues. Progress in the synthesis of successful nanoparticles (NPs) relies on developing an accurate method for the design of these submicron drug delivery systems. At the moment, simulation approaches are extremely useful for performance evaluation of materials and also for prediction of desired properties in various engineering issues. Additionally, simulations suggest an accurate description of the mechanisms behind many phenomena, which is relatively difficult to explain by experiment outcomes. Also, employing mathematical models prior to conducting experimental evaluations not only reduces the cost of assessment but also eliminates the need for needless analysis, which basically takes a lot of time. Finally, it is undeniable that simulation techniques assist in minimizing the health hazards associated with working with potentially harmful chemicals^8,9^.

To date, researchers have reported various aspects of polymeric NP interactions with biological membranes^10^, drugs^11–13^, peptides^14,15^, BSA^16^, and proteins^17^. Among different agents, polymer-drug interactions, especially anticancer drugs, have attracted attention recently^11,18^. For instance, Razmimanesh and her colleagues investigated the loading efficiency of chitosan for gemcitabine (chemotherapeutic) and found that the maximum loading of the drug occurred at 40% of the drug concentration, and in the absence of water molecules, the drug molecules were located at a shorter distance from chitosan, which led to higher drug loading efficiencies^13^. In another study, the interaction of poly(acrylic acid) with doxorubicin (DOX) was investigated at various pH values as model gastric and intestinal fluids. The results showed that the diffusion coefficient of DOX decreases with an increase in pH due to an increase in the ionic complexation of polymers with DOX^19^. Karatas et al. illustrated that a combination of PLGA with montmorillonite (MMN) could serve as a nanoscale delivery system for curcumin (an anticancer drug)^20^. The results revealed that without MMT, PLGA encapsulates curcumin; however, the presence of MMT improves drug release due to the increased diffusion rate.

However, there are a few reports on simulated PLGA-PEG combinations, which are very common in experimental assessments due to their properties as a result of a combination of hydrophobic and hydrophilic parts. For example, Jafari et al. considered the influence of PEG chains in various combinations with PLGA polymers on the stability and aggregation behavior of micelles^21^. Their results revealed that regardless of the sequence of polymers, the aggregation behavior of the micelles had no significant change. In another study, MD investigation of PLGA-PEG NPs loaded with a hydrophobic model drug (itraconazole) explained the experimentally observed low drug loading content^22^. Interestingly, it was shown that the phenomena are due to the positioning of the drug in the water-nanoparticle interfaces because of unfavorable interactions of the drug and hydrophobic PLGA core.

Our recent work addresses the impact of both polymers and targeting ligands on NP properties^23^. In this paper, we optimized the ligand (riboflavin or vitamin B2, RF) density on polymeric nanoparticles to achieve a stable structure. Outstandingly, the calculated gyration radius (R_g_) verified the trend observed in the experimentally measured hydrodynamic diameter (D_H_) size of nanoparticles. The results confirmed that as the minimum size of R_g_ and D_H_ occurred in the 20 and 10 wt% PLGA-PEG-RF polymer chains in the nanoparticles, these compositions formed the most stable particles for PLGA _3 kDa_-PEG_2 kDa_ and PLGA _4.5 kDa_-PEG_2 kDa_, respectively. The accuracy of the simulations encouraged us to predict the interaction of the anticancer drug with designed polymeric NPs. As a follow-up study, docetaxel (DTX) and 4 another drugs compared was introduced as a model chemotherapeutic to the polymeric solution before nanoprecipitation to mimic the real drug loading process. This study contain All Atom and Coarse Grained simulations and Dynamic Light Scattering (DLS) and Zeta Potential Experimental tests for different drugs at the best concentration and all of the result show the best stability of the DTX drug.

## Material and methods

### Polymer designation

The polymers used in the current simulations are based on previous experimental and computational approaches. In brief, polymers are designed as nonconjugated (PLGA-PEG) and RF-conjugated (PLGA-PEG-RF) polymers with different molecular weights (see detail of polymers structures in the reference^24^). For each simulation case, a simulation box includes a combination of the PLGA-PEG-RF and one of the PLGA-PEG strands to investigate the influence of the moiety on the physicochemical properties of NPs.

It is worth mentioning that chains have different repeating units. In more detail, the repeating unit of the PLGA segment of the PLGA_4.5 kDa-PEG2 kDa_ chain consists of two lactic acids with one glycolic acid. However, PLGA_5kDa_-PEG_3.4 kDa_-RF and PLGA_3kDa_-PEG_2kDa_ have one glycolic unit that respected each lactide unit. The polymers are designed due to their real structures that have been used in the experimental environment.

### Simulation parameters

We designed molecular dynamics-based systems to simulate the real self-assembly process using GROMACS software. The simulation approach has been described in more detail previously^23^. The structures of docetaxel, PLGA-PEG, and PLGA-PEG-RF were constructed using Quantum espresso software. The polymer chains had 20, 26, and 30 nm lengths for PLGA_3kDa_-PEG_2kDa_, PLGA_4.5 kDa_-PEG_2kDa,_ and PLGA_5kDa_-PEG_3.4 kDa_-RF, respectively.

Afterward, the chains were sequentially optimized using Avogadro and HyperChem software’s UFF and OPLS-AA force fields. In the next step, optimizations were carried out using Quantum espresso software with the ONIOM method, which includes three layers: high (b3lyp and basis set 6–31 +  + G (d,p)), medium (b3lyp and basis set sto-3 g), and low (of PM6). All three layers contain DFT and semiempirical computational algorithms. At the main optimization step, we used GROMACS 2019.3 (OPLS-AA force field^23,25^) in the EM (10 kJ/mol/nm minimum force), NVT (500 ps), NPT (500 ps), and then MD (100 ns) simulations in 2 fs time steps. All cases with varying numbers of PLGA-PEG:PLGA-PEG-RF polymer strands contained 30,600 water molecules (SPC/E water model) in boxes of 3 × 3 × 30 nm^3^.

Simulations were accomplished to form NPs from copolymers in a cubic box with 8 and 8.5 nm lengths for PLGA_3kDa_-PEG_2kDa_ and PLGA_4.5 kDa_-PEG_2kDa_, respectively, to keep the polymer concentration at 10 mg/ml. To achieve a constant drug concentration in both cases, 3 mg/ml, 9, and 12 DTX molecules were added to the simulation boxes based on _PLGA 3 kDa-PEG2 kDa and PLGA 4.5 kDa-PEG2 kDa_, respectively. The cut-off radius was adjusted to 1.4 nm for the van der Waals and Colomb interactions. We used the Colomb energy algorithm, particle mesh Ewald (PME). The MD simulation step was carried out with the isotropic Parrinello–Rahman algorithm^26^ at 1 bar and with a nose hoover (velocity-scaling algorithm in NVT and NPT) at 300 K. The constraint algorithm was based on the LINCS algorithm used only for hydrogen bonds^21,22,27^. The partial atomic charges of the structures are calculated using the same force field (Fig. S1 and Table S1). Copolymers equilibrated in a simulation cubic box with 8 and 8.5 nm lengths for PLGA 3 kDa-PEG2 kDa and PLGA 4.5 kDa-PEG2 kDa, respectively, to keep the concentration at 10 mg/ml. All optimizations were carried out in 100 ns (or 5 × 10^7^ numbers of 2-fs time steps) at 300 k. The assembly of the ten polymers was simulated in similar boxes with a course of 100 ns^28,29^.

All atom simulations for different drugs done at the optimum state of PLGA-PEG-Rf concentration that is 20%. These simulations repeated like another all atom simulations and the drugs Parametricized like DTX parametrization. And Coarse Grained simulations repeated for 3 best drugs at 1100 ns and 20 fs time steps with Martini ff. parametrization of polymers and drugs was manualy and was according to the Martini ff article^30^.

### Calculation of drug loading and encapsulation efficiency

We determined the drug loading and encapsulation efficiency (EE) in the NPs. Drug loading is defined as the total mass of drugs in the total mass of NPs. The EE (%) is also defined as the final amount of drug present in the formed NPs with respect to the initial amount of drug used for studies:

1 $$ drug\;loading \left( \% \right) = \frac{total\;mass\;of\;drug\;in\;nanoparticle}{{total\;mass\;of\;nanoparticle}} \times 100 $$

2 $$ EE\left( \% \right) = \frac{total\;mass\;of\;drug\;entrapped\;in\;nanoparticle}{{Initial\;mass\;of\;drugs\;used\;in\;simulation}} \times 100 $$

### Gibbs free energy calculation

The Umbrella sampling technique was used for the Gibbs free energy calculations. Umbrella simulation input structures are NPs formed at the output of the MD simulation. This method was performed for all PP:PPR ratios, including two simulation steps. First, pull code was used to separate one of the 10 polymers from aggregation. In the second step, 100 configurations are extracted from pull code simulation. For each configuration, 1 ns simulation is performed. After applying pull code for the polymer strand, it restrained at increasing center-of-mass (COM) distance from polymer strands, leading to the generation of various configurations for each location. The PMF curve is extracted in the restraint stage using the polymer strand positions to the COM. In other words, the integration of PMF corresponds to the series of configurations. Finally, Gibbs free energy is obtained by WHAM^31,32^ analyses on all configurations. The WHAM analysis method is a very powerful technique based on estimating the statistical uncertainty of the probability distribution provided by the umbrella method^33,34^. As a result, the smallest uncertainty can be computed by the PMF results. The dimensions of the simulation box are 10 × 10 × 30. Energy minimization is applied to the NPs, and then NPT equilibrium is performed on the molecules. The temperature and pressure algorithms are Berendsen^35^. The temperature and pressure algorithms are Nose–Hoover and Parrinello-Rahman, respectively. Finally, the Gibbs free energy for each simulation was calculated by WHAM analysis of the 100 configurations.

### Experimental method

Dynamic light scattering analysis (DLS) was done, by a VASCO Cordouan Technologies, to measure nanoparticles size distribution. More ever, SZ-100 HORIBA Zeta Potential analyzer was employed to check stability.

### Experimental validation

In a molecular dynamics simulation system, if an experimental test or another simulation can be produced with a similar force field and simulation method, it can be said that that force field and that method are intended for materials and nanostructures, It is valid and trusted.

In this regard, five experimental simulation systems were produced by the method and force field presented in the methodology section and presented in Fig. 2. Figure 2I is from an article presented in the journal RSC^24^, in which the nanoparticle size with different percentages of PLGA-PEG-Rf polymer concentration was investigated during 6 different simulations and the exact same trend reported in the diagram was obtained. This simulation is from the Fig. 5-ci diagram in an article by Rezvantalab et al. In this simulation, polymers with longer chain lengths, namely PLGA _4.5 kDa_-PEG_2 kDa_ were used.

In (Fig. 2II), which is related to an ACS paper^36^, the size of nanoparticles of experimental work in Fig. 6 was modeled from the paper of rezvantalab et al. By simulation work. Is modeled, namely PLGA _3 kDa_-PEG_2 kDa_. The size data of the synthesized nanoparticles were completely consistent with the data obtained from the analysis of the gyration radius.

The third validation is shown in (Fig. 2III)^37^, from the journal Teylor and Francis. In this paper, Khaledi et al. Examine the PLGA-PEG-PLGA and 5-Fluorouracil and Chrysin polymer delivery drag. Figure 4-b This paper examines the percentage of Chrysin release at different temperatures and pHs. This study has been calculated at different times, but from 60 to 100 h the release percentage has been stable. The final stabilized value of this percentage of release was after 100 h, and in the simulation performed, the percentage of release was compared with this final value. Finally, the data obtained from the simulation and experimental test were very well matched, and both sets of data went through the same process with very good accuracy, which is a validation of the method and force field used in this paper. The release of drugs percents in simulation and experiment systems, computed by Eq. (3).

3 $$ Release\;of\;drugs \left( \% \right) = 1 - drug\;loading\left( \% \right) $$

In Fig. 2IV, the DLS sizes shown in Fig. 7 of Lopez-cano et al.'s paper are modeled using this method and force field. This article, which is an MDPI article^38^, deals with the treatment of retinal degenerative diseases with the help of PLGA-PEG-PLGA hydrogel. In Fig. 7 of this paper, the size of micelles with different concentrations of DX, Idebenone, TPGS and KT were measured by the DLS experimental tests and compared with the simulation data. A very similar process to the data shows that the simulation method and the force field used are very suitable for this type of polymer.

**Figure 1:**
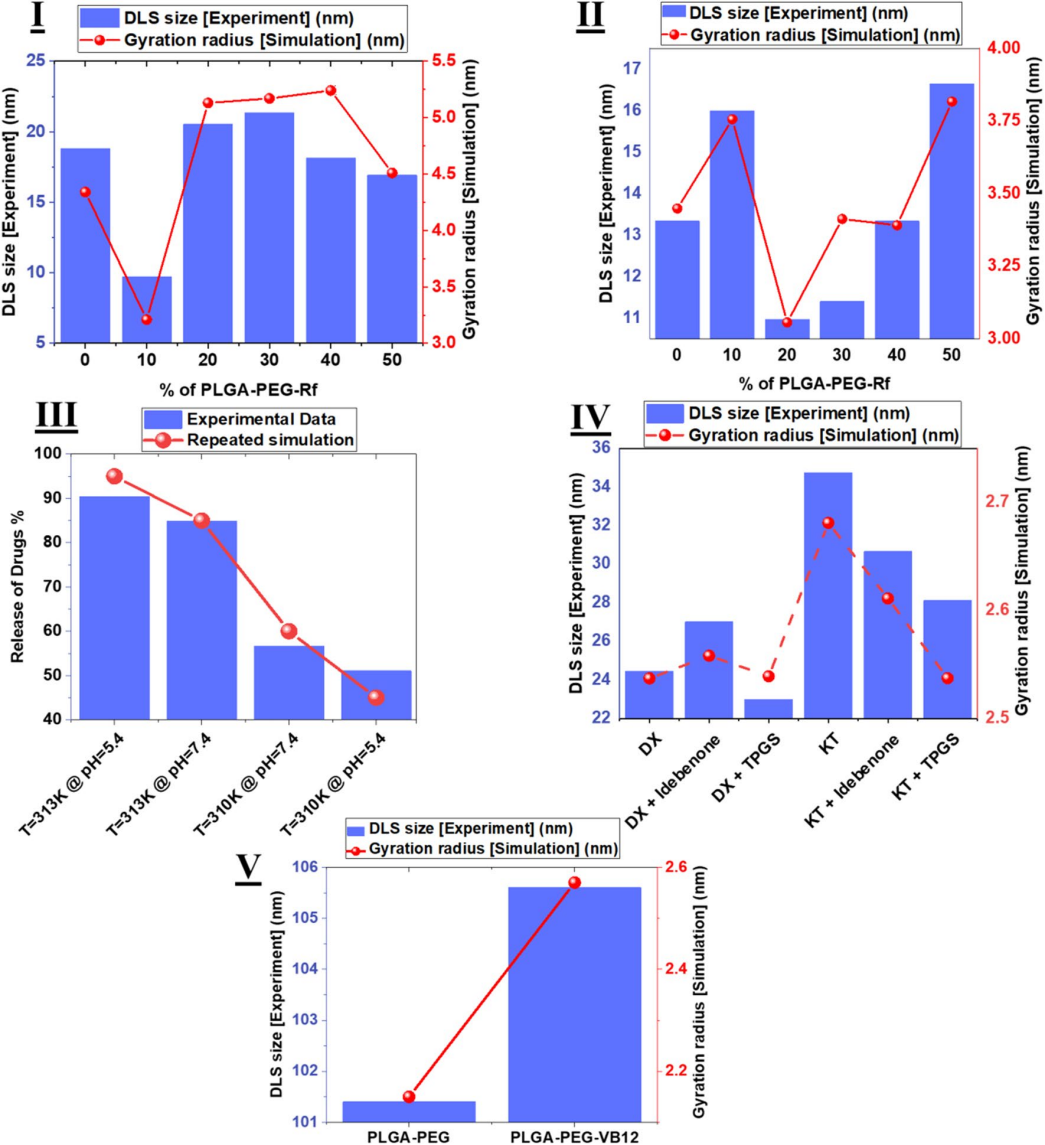
Model of experimental validations. (**I**) and (**II**) Comparison of simulation data and laboratory data obtained from Dynamic Light Scattering (DLS) test for simulations of polymers with PLGA _4.5 kDa_-PEG_2 kDa_ and PLGA _3 kDa_-PEG_2 kDa_ chains, respectively. Which have been published in RSC and ACS journals, respectively. (**III**) This graph is also a comparison of the release rates for PLGA-PEG-PLGA nanostructures and Chrysin drug at the different Temprature and pH, which is the subject of an article in the journal Teylor and Francis. This data is also compared in both simulation and experimental modes. (**IV**) These data are also related to the comparison of simulation data and DLS experiment tests for PLGA-PEG-PLGA hydrogels and different concentrations of DX, Idebenone, TPGS and KT drugs, which was published in the journal MDPI. (**V**) This sample is also related to the comparison of the size of micelles obtained from DLS experimental tests and simulations performed. In these two simulations, the size of PLGA-PEG and PLGA-PEG-VB12 micelles are compared.

**Figure 2:**
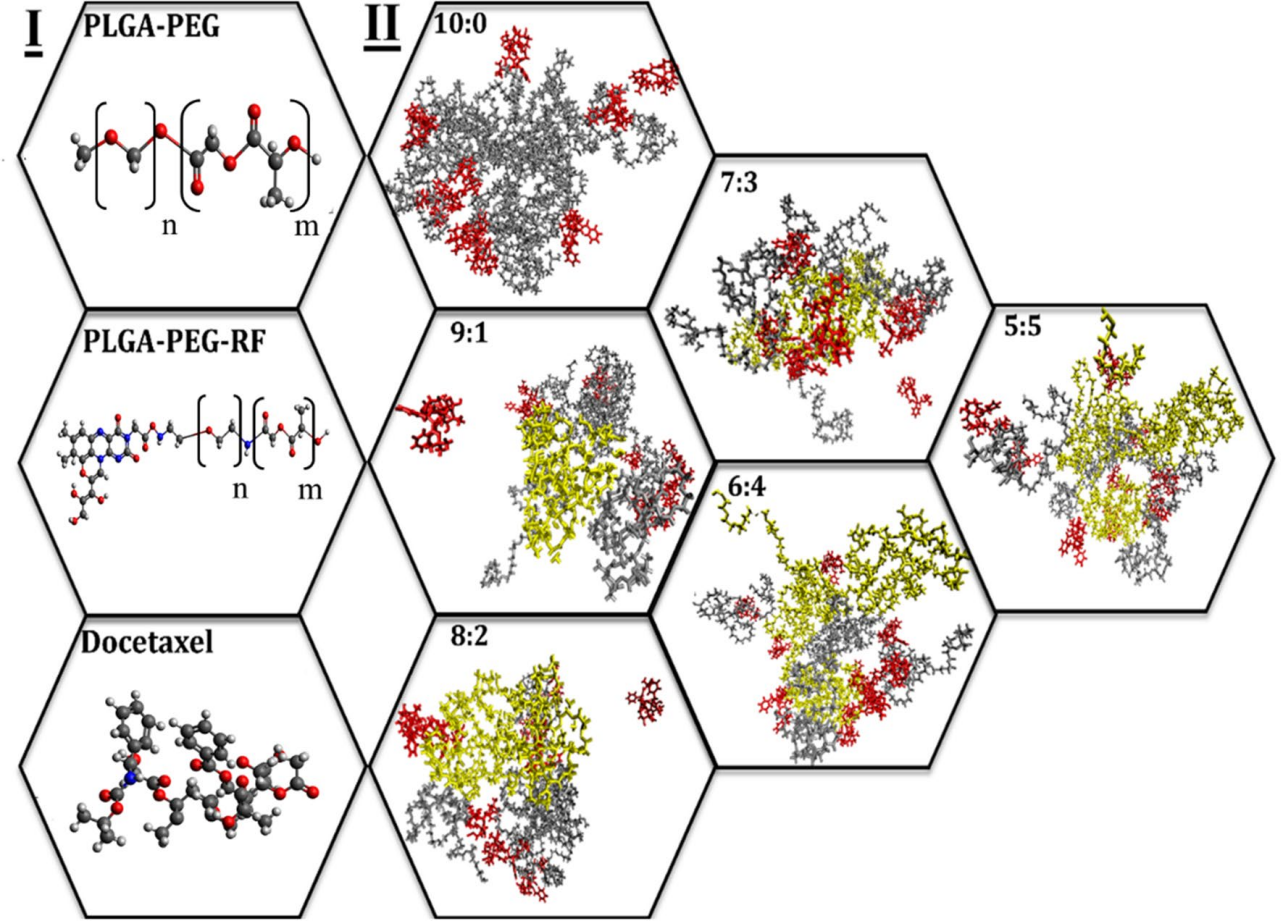
Graphical presentation of simulations; (**I**) Structure of docetaxel, PLGA-PEG, and PLGA-PEG-RF (*Nitrogen in the structure is blue, Carbon is silver, Oxygen ins red and Hydrogen is white*). (**II**) Drug-loaded RF-conjugated NPs formed within a 100 ns timespan at different PP:PPR ratios. PLGA-PEG, PLGA-PEG-RF, and drug molecules are represented in gray, yellow, and red, respectively.

Figure 2V of the paper also compares the size of PLGA-PEG and PLGA-PEG-VB12 micelles in both experimental and simulation modes. These tests are presented in Fig. 1b of Elsevier's article^39^, published by Chen et al. In this paper, the DLS sizes have a very good similarity with the gyration radius in the simulation, which is quite noticeable in (Fig. 2V) The ligand used in this article and the ligand used in this article are both from the B vitamin family.

In general, according to these studies and due to the same process of data obtained from laboratory tests and simulations, it can be easily concluded that this simulation method and this field of force are very suitable for polymers and materials used. And the data reported from this simulation in this article are completely valid data.

## Results

In our previous paper^24^, we showed that for two polymers with different molecular weights, the stability of the NPs is different and achieved in different PP:PPR (PLGA-PEG:PLGA-PEG-RF) ratios (Fig. 2I and II). For the main polymer in the NPs with two PLGA molecular weights (3 and 4.5 kDa), the PEG chain molecular weight was considered to be 2 kDa, since it was shown that PEG chains with 2 kDa are the most efficient chain length in the interaction with cells and targeting the receptors^40^. To participate in 10 mg/ml polymer chains with 3 mg/ml drug in water, 10 polymer chains were added to the simulation box. However, polymers have various molecular weights, which leads us to use simulation boxes of different sizes. Moreover, to keep the drug concentration at 3 mg/ml in both cases, varying numbers of DTX molecules were incorporated. For PLGA_3kDa_-PEG_2kDa_ and PLGA_4.5 kDa_-PEG_2kDa_ simulations, the proper number of anticancer molecules, 9 and 12 DTX molecules, are used, respectively. It is worth mentioning that simulations are carried out in the presence of water molecules to mimic nanoprecipitation under real conditions. The videos attached as supplementary material display the nanoprecipitation process and location of drugs and polymers in the final NP structures.

### Stability of NPs

**Figure 3:**
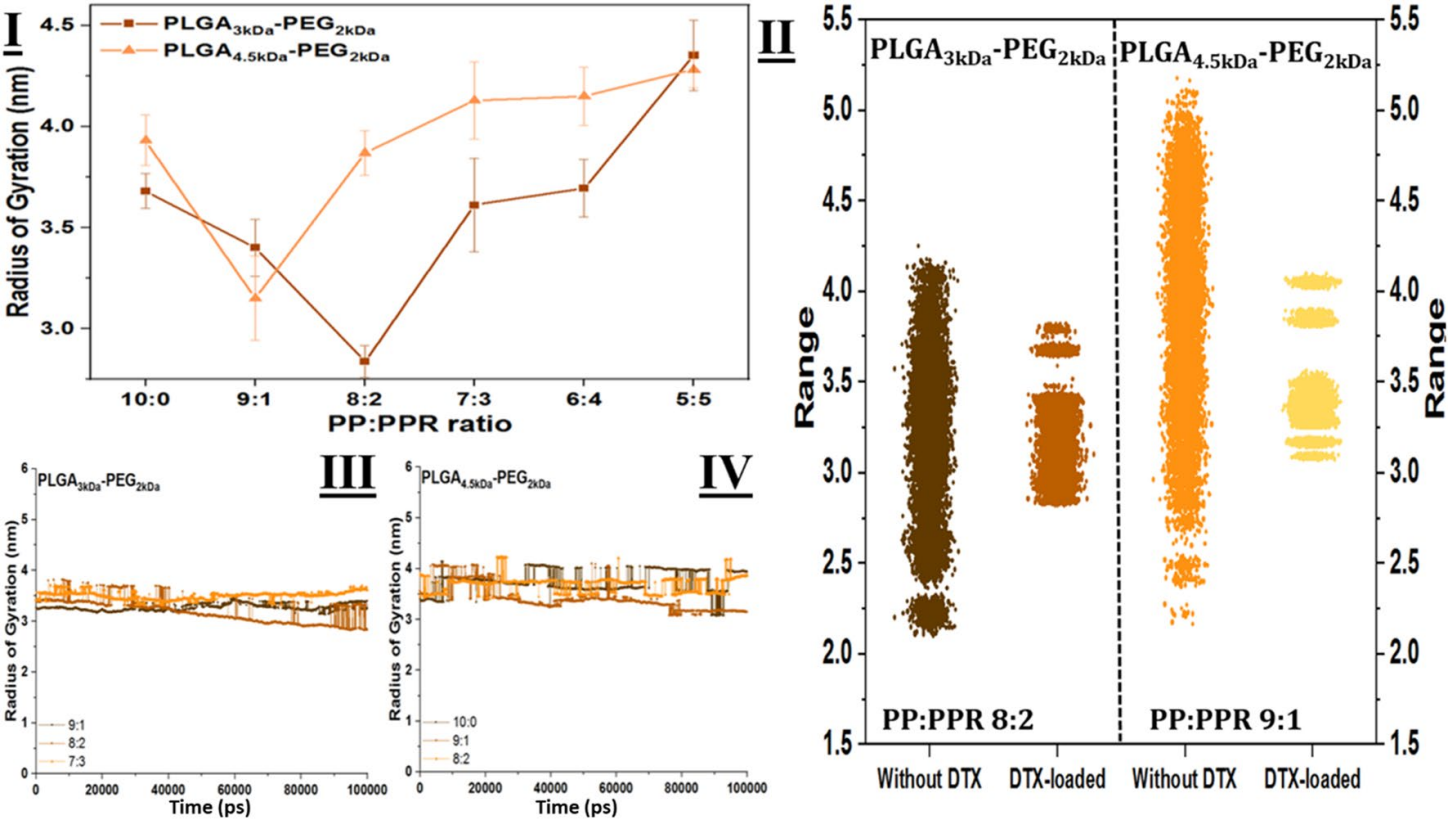
Gyration radius of nanoparticles. (**I**) Final R_g_ of NPs based on PLGA3 kDa-PEG2 kDa and from PLGA4.5 kDa-PEG2 kDa in the combination with varying ratios of PLGA4.5 kDa-PEG2 kDa-RF in the composition. As is clear, stability changes depending on the molecular composition of repeating monomers in the structure of the basic polymer strand. (**II**) The addition of DTX molecules to the NPs affects the fluctuations of NPs during assembly. (**III**) and (**IV**) R_g_ fluctuations for drug-loaded RF-conjugated NPs vs. time reveals the stability and lower range of variations.

The results obtained in the simulation set display a similar trend to the results of our last article, which further confirms the previous results. Figure 3I reveals the final radius of gyration (R_g_) of the NPs based on both polymer chains. The minimum of the plots occurs in the same compositions that were previously detected as the stable compositions of the RF-conjugated NPs. In more detail, using only 2 (out of ten total polymers) RF-containing chains in the composition of NPs based on PLGA_3kDa_-PEG_2kDa_ provides the smallest (2.85 nm) particle among its peers. Moreover, it is the smallest of all samples, whereas the other sets of NPs based on PLGA_4.5 kDa_-PEG_2kDa_ have the smallest (3.15 nm) sample at PP:PPR 9:1 ratio. We can contribute to the observed outcome of the nature of polymers since the PLGA_4.5 kDa-PEG2 kDa_ chains have a larger and higher number of repeating units. Additionally, to keep the same drug concentration for both categories in the simulation of the assembly process, we used 3 more DTX molecules in comparison with the particles based on the PLGA_3kDa_-PEG_2kDa_ NPs. All mentioned reseasons add up to the final R_g_ values that are larger for PLGA_4.5 kDa-PEG2 kDa-_based assemblies.

In the following step, we compared the Rg oscillations for both detected minimum samples to consider the effect of drug molecules. Figure 3II demonstrates the range of oscillations with and without anticancer agents in the NP compositions. However, for unloaded NPs based on _PLGA 3 kDa-PEG2 kDa_ at PP:PPR 8:2, the final R_g_ was smaller (2.50 nm). However, its fluctuations during the simulation reveal that the loaded particle is more stable. A similar explanation is valid for the DTX-loaded particles based on _PLGA 4.5 kDa-PEG2 kDa_. Fig. S2 represents the fluctuations of R_g_ for both sets of NPs over the course of the simulation.

To avoid cluttered diagrams, we present only a few samples for each category, but the rest also have similar trends. For the smaller polymer (Fig. 3III), in the case of a PP:PPR ratio of 8:2, R_g_ has an initial value of approximately 3.4 nm, which increases up to 3.8 nm during NP formation and finally drops to approximately 2.8 nm. Although the other two samples also fluctuate during nanoprecipitation, samples 9:1 and 7:3 start at 3.41 and 3.27 nm, respectively, and end up at even higher values (3.6 nm and 3.4, respectively). This confirms that the 8:2 sample reaches stability by the end of the simulation. A similar trend is also observed for PLGA_4.5 kDa_-PEG_2kDa_ (Fig. 3IV). The 9:1 system’s R_g_ decreases to 0.5 nm less than the initial value (3.65 nm), while for the 10:0 sample, _the_ final Rg value is higher than the initial value, and the 8:2 sample has almost the same R_g_ amount as the starting point. Remarkably, the results confirm the stability obtained previously.

To better assess NP stability, it is important to consider the strength of intramolecular interactions. The total energy of a system is equal to the bonded energies plus nonbond energies^11^. Nonbonded energies are equal to the sum of the van der Waals (vdW), electrostatic interactions, and hydrogen bond energy. Therefore, it can be said that the total enthalpy changes for our systems (in which there is no bonded interaction) are equal to the sum of the changes of the vdW, electrostatic, and hydrogen bonds. The energy of vdW and electrostatic interactions is calculated via the Lennard–Jones equation and Colon law, respectively. The total energy is the sum of both of them that are dominant in the self-assembly process^41,42^.

**Figure 4:**
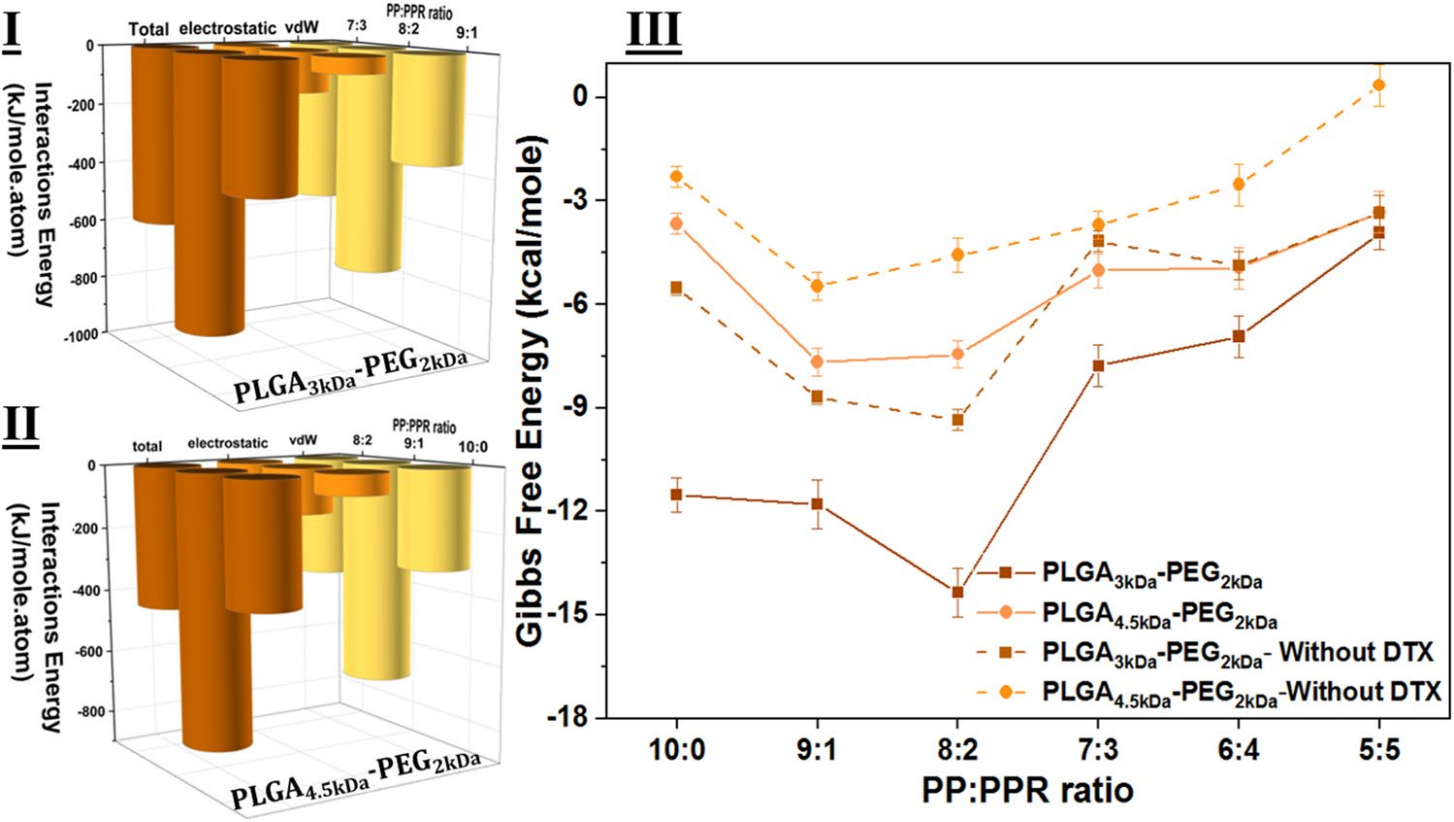
Energy assessment of simulations. (**I**) and (**II**) Formation enthalpy of particles based on PLGA¬3 kDa-PEG2 kDa and PLGA¬4.5 kDa-PEG2 kDa, respectively. It is obvious that for both sets of NPs, the vdW interaction is the dominant interaction in the assembly of polymers and drugs. (**III**) absolute criteria of stability based on Gibbs free energy. Drug loading into NPs lowers the Gibbs energy level, which means that particles are more stable.

Due to the different number and composition of repeating units in utilized polymer strands, the calculated energies of simulations are different. Although all simulated cases contain ten copolymer chains, due to the longer chain of PLGA-PEG-RF, more RF-conjugated polymer in the composition results in a higher energy level. To avoid the misinterpretation of the results, we normalized the results regarding the atom numbers. Figure 4I and II demonstrate the results of the computation. In an attempt to provide clear plots and easy-to-understand, in each category, three analyses are presented. The plots reveal the energy of _PLGA 3 kDa-PEG2 kDa and PLGA 4.5 kDa-PEG2 kDa_ polymers at PP:PPR ratios of 8:2 and 9:1, respectively, reaching the maximum attraction energy, which indicates that at these densities, NP adsorption is higher than that of their peers in each group. It is worth mentioning that, as in other reports^20^ on the self-assembly of PLGA-based NPs, vdW interactions are the dominant interactions. It can be concluded that the vdW interactions are responsible for the attraction and, consequently, the shrinkage of Rg at 20% and 10% RF-conjugated NPs.

Gibbs free energy is the absolute criterion for the determination of stability. In this line, we performed Gibbs free energy calculations using the Umbrella sampling technique to provide reliable and precise evidence of stability. Figure 4III shows the results of Gibbs free energy calculations for NPs with and without DTX molecules. Both dotted lines represent the cases without drugs. As previously stated in Fig. 3II, R_g_ fluctuations in a broad range of unloaded samples show instability. Here, Fig. 4III demonstrates that drug contents pull down the energy level of particles and improve their stability. The minimum free energy occurs in the same cases for each category. In other words, drug content improves the stability of particles; however, the most stable NPs have the same amount of RF-conjugated polymers as without drugs.

From Gibbs free energy calculations, it can be observed that RF-conjugated NPs based on _PLGA 3 kDa-PEG2 kDa_ are more stable than PLGA 4.5 kDa-PEG2 kDa-based particles. For instance, at a PP:PPR 8:2 ratio, the free Gibbs free energy level is − 14.35 kcal/mole for the PLGA_3kDa_-PEG_2kDa-_based particle. On the other hand, with the same PP:PPR ratio based on PLGA_4.5 kDa_-PEG_2kDa,_ the energy level rises up to − 7.44 kcal/mole.

### Nanoparticles morphology

**Figure 5:**
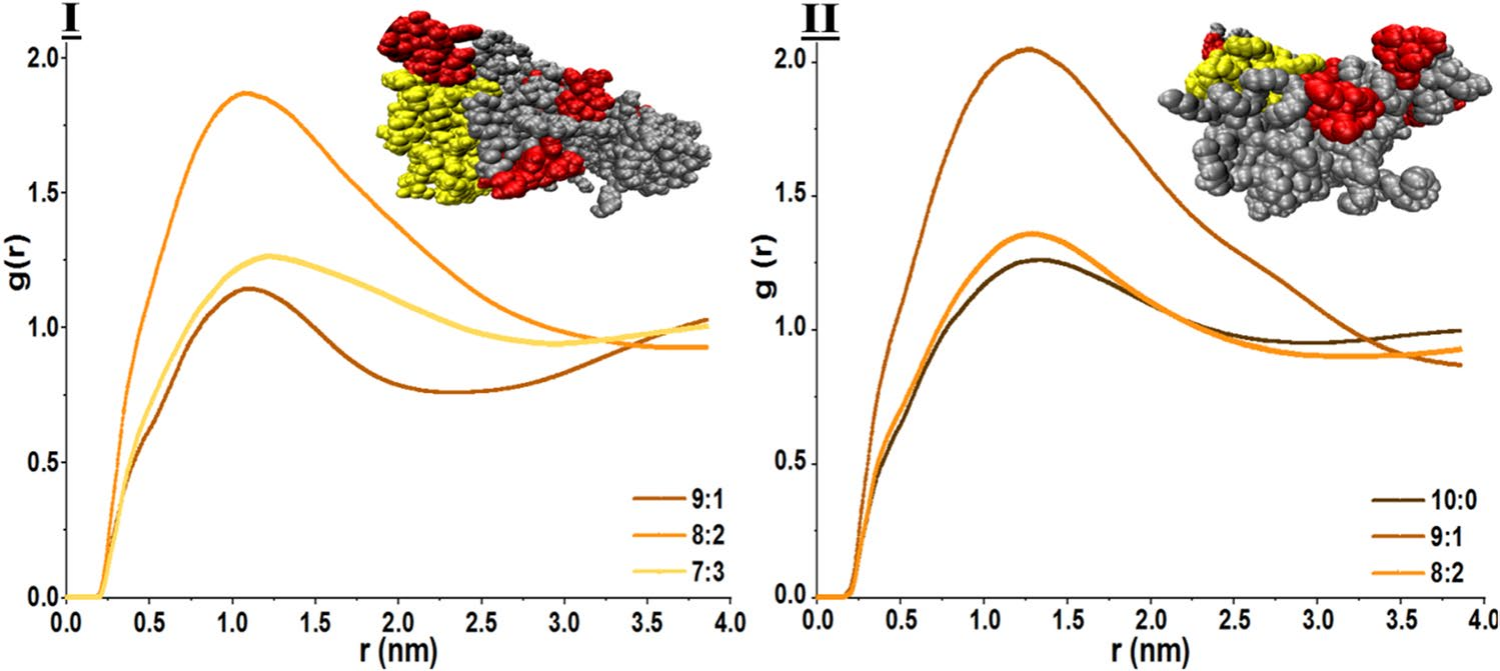
Morphology of stable NPs. Radial distribution function (RDF) of NP around the drugs. (**I**) Radial distribution function (RDF) of NP around the drugs for PLGA_3kDa_-PEG_2kDa_ at the 8:2 ratio together with a snapshot of the final particle verify the highest drug loading and encapsulation. with 5 antitumor molecules in the mass and 3 on the surface. (**II**) RDF plot for the NPs based on PLGA_4.5 kDa_-PEG_2kDa_ declares the highest polymer distribution around the DTX molecules at PP:PPR 9:1. A snapshot of the polymeric nanocarrier also verifies the results. Notably, all RF-targeted polymer chains are located at the surface.

The main aim of the current study was to investigate drug loading in riboflavin-conjugated NPs using simulation-based methods. After evaluation of the stability of the sample and detection of stable samples, in the next step, we assessed the drug loading and entrapment results (Table 1). First, we noticed that some drug molecules were encapsulated by the PLGA core of the NPs. However, there are few DTXs in the vicinity of NPs with water molecules. It was shown in PLGA-based NPs that drugs are mostly located at the interface of polymeric NPs with water^22^. We also perceived DTX molecules in the NP vicinity as entrapped drugs. In this line, for PLGA_3kDa_-PEG_2kDa_ although sample 8:2 has 5 drug molecules in the center of particle mass, considering drug molecules in the district of the shell (Fig. 5), in total it has the highest drug loading and entrapment efficiency. In other words, samples 8:2 and 7:3 have similar EE (%), but considering drug loading results, it can be concluded that the 8:2 composition (as the most stable case) has the highest drug loading and encapsulation efficiencies. Similar outcomes were observed for the NPs based on the PLGA_4.5 kDa-PEG2 kDa_ polymer. This means that the detected stable sample has the highest drug loading and encapsulation efficiencies.

**Table 1:** Final results of drug entrapments in the simulated NPs.

PLGA_3kDa_-PEG_2kDa_ samples	7:3	8:2	9:1
Drug loading (%)	18.73	19.86	18.99
Encapsulation efficiency (%)	77.78	88.89	88.89
PLGA_4.5 kDa_-PEG_2kDa_ samples	8:2	9:1	10:0
Drug loading (%)	22.53	22.55	21.29
Encapsulation efficiency (%)	75	91.67	83.33

Polymeric NPs prepared from variable ratios of _PLGA 5 kDa-PEG 3.4 kDa_ and PLGA_4.5 kDa-PEG 2 kDa_ also displayed interesting results. All have the same number of drugs in the center of mass, and with the same reasoning, EE efficiency surges to the highest amount in the 9:1 proportion of nontargeted and targeted polymers. Among all other samples, PP:PPR 10:0 for PLGA_4.5 kDa-PEG2 kDa_ showed the lowest drug content. The same hydrophobic interactions are the main cause of improved EE efficiency. With another 10% increase in the PLGA-PEG-RF content, longer PEG chains are present on the surface, which can interrupt hydrophobic interactions and cause a reduction in the drug upload in the micelle. Close inspection of NPs with surrounding water reveals that NPs are hydrophobic and that all water molecules are located outside of the total mass (Fig. S3). Furthermore, DTX molecules have more interactions with nontargeted molecules, and most of them are entrapped among PLGA-PEG chains.

The radial distribution function (RDF) of polymer chains around the drug molecules is analysed and plotted in Fig. 5. The interaction between DTX and polymer chains is illustrated by the peaks at approximately 1 nm (Fig. 5) for all systems of _PLGA3 kDa-_based NPs, with the highest peak for the 8:2 model. It can be concluded that the strongest interaction between drugs and polymers belongs to the composition containing 20% of the targeted polymer. The highest peak for the PLGA_4.5 kDa_ samples also occurred in the 9:1 model. Additionally, the height of the peaks increases, and the position of the peaks moves nearly 1.25 for the larger polymer. Comparing results declares that not only are 8:2 and 9:1 samples the stable models for PLGA_3kDa and_ PLGA_4.5 kDa,_ respectively, but they also have the strongest interactions with drug molecules with higher entrapment efficiencies.

### Comparing of different drugs at the stable concentration

#### All atom simulations (Comparing 5 drugs at the 100 ns)

**Figure 6:**
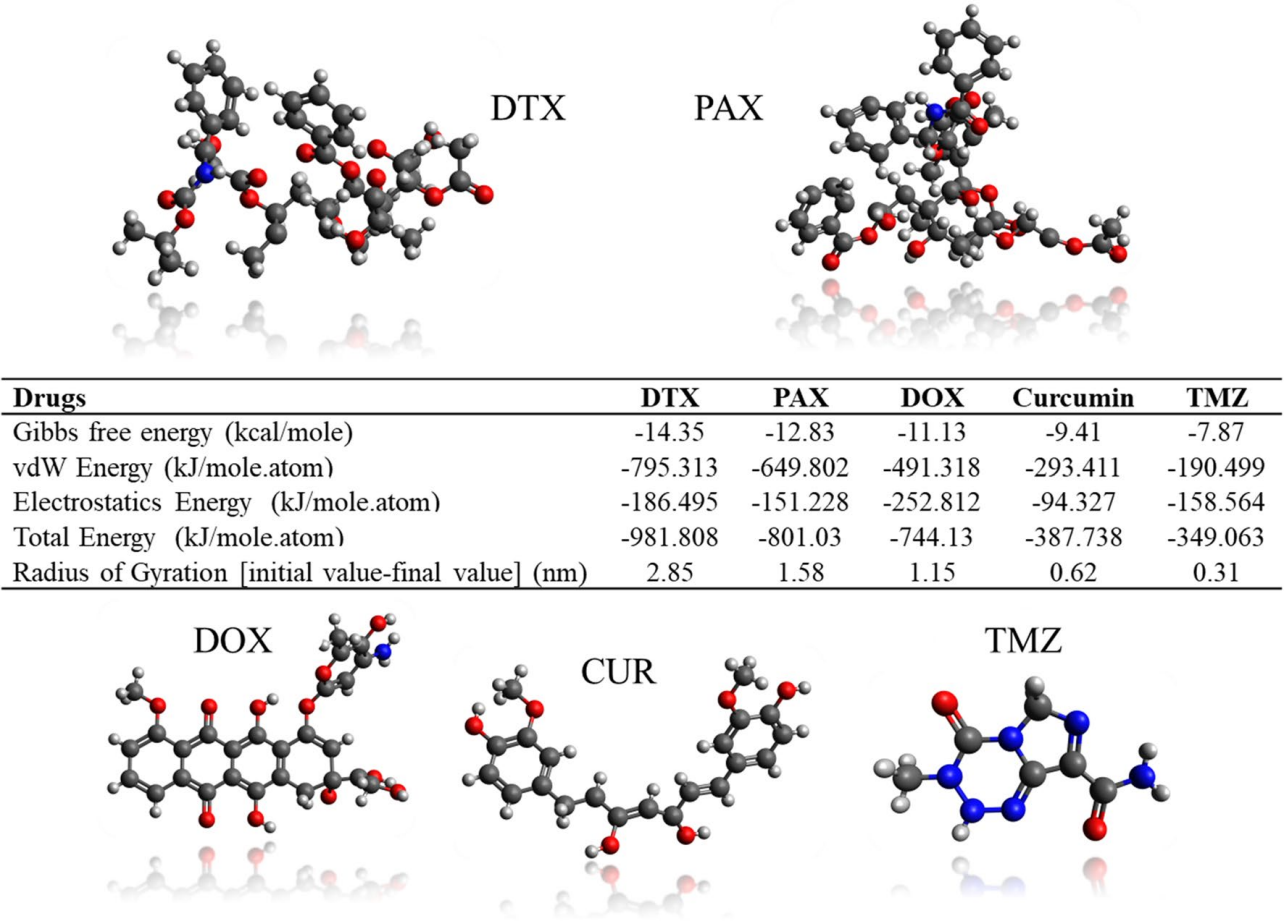
In this figure has been shown Gibbs energy analysis, van der Waals and electrostatic energy analysis, total energy analysis, and gyration radius difference analysis for the DTX, PAX, DOX, CUR and TMZ drugs that simulaed in 20% cocentration of PLGA-PEG-Rf. These simualation are All atom simulations at 100 ns. (Nitrogen in the structure is blue, Carbon is silver, Oxygen ins red and Hydrogen is white).

In Fig. 6 of this paper, for the optimal state of PLGA-PEG-Rf polymer concentration, which corresponds to the PLGA_3kDa_-PEG_2kDa_ chain length with a concentration of 20% of PLGA-PEG-Rf polymer, simulations with 5 different drugs are shown.

The drugs used were DTX, Paclitaxel (PAX), Doxorubicin (DOX), Curcumin (CUR) and temozolomide (TMZ), respectively. These simulations have been investigated by Gibbs energy analysis, van der Waals and electrostatic energy analysis, total energy analysis, and gyration radius difference analysis. Comparing all these drugs, we can see that DTX is the best drug for nanocarriers. According to the general specifications presented in Fig. S4, it is easy to understand that DTX drug, having the highest molecular weight compared to other drugs and the high number of rings in its structure, forms a very large van der Waals formation compared to the other 4 compared drugs. After DTX drug, PAX has the best conditions among drugs.

This high amount of van der Waals energy causes the total energy between the nanocarriers and the drugs to be higher for DTX, and therefore the drug has a more negative Gibbs energy, a larger radius difference and stronger aggregation, and therefore more stable conditions for transmission. In the body.

#### Coarse grained simulations (Comparing 3 drugs at the 1100 ns)

**Figure 7:**
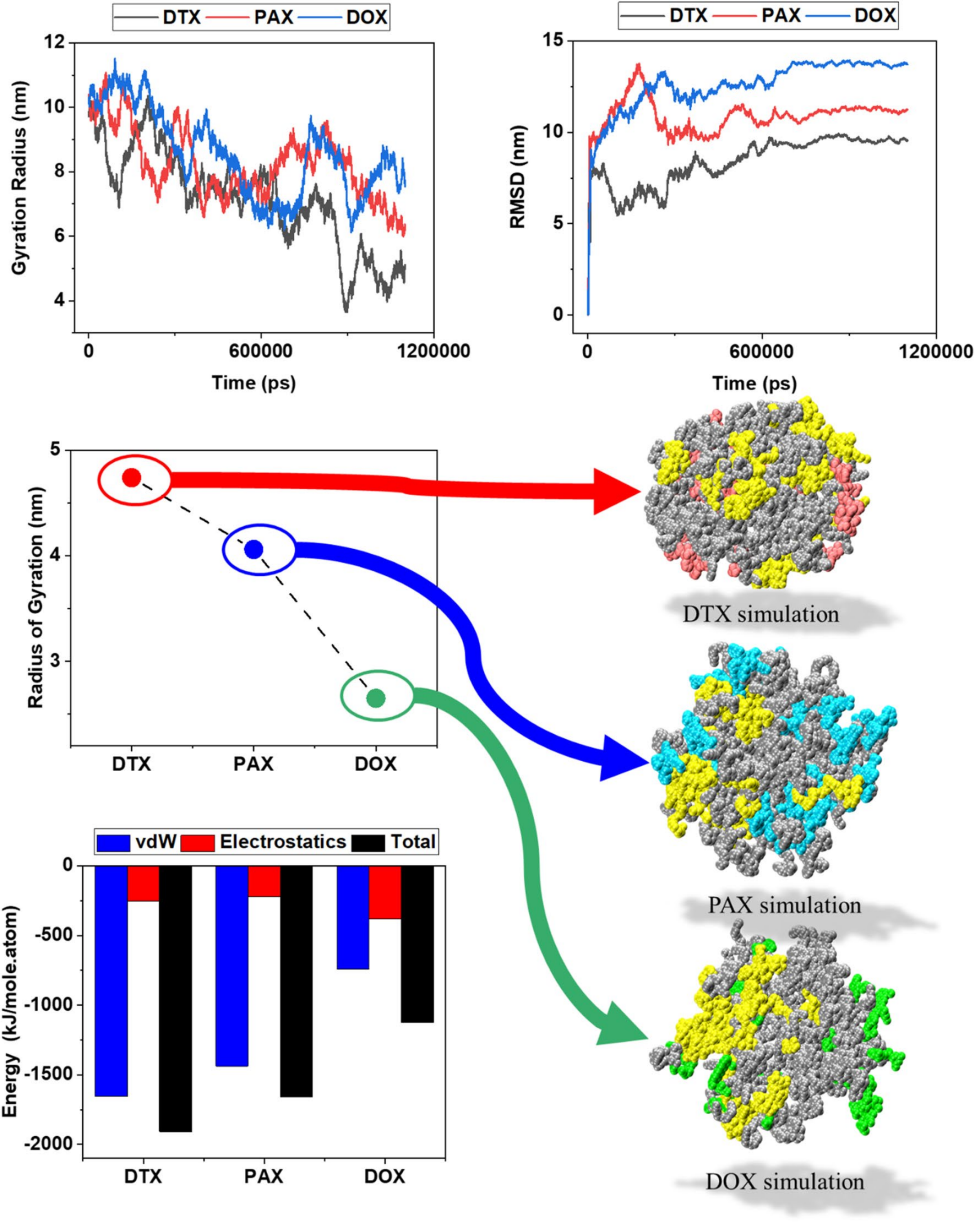
In this figure has been shown van der Waals and electrostatic energy analysis, total energy analysis, and gyration radius difference initial and final point, gyration radius at the periodic of time and RMSD analysis at the period of time for the Coarse Grained simulation at 1100 ns. (PLGA_3kDa_-PEG_2kDa_ polymers in the simulation pictures are silver, PLGA-PEG-Rf polymers are yellow, DTX drugs are red, PAX drugs are blue and DOX drugs are green).

For the 3 drugs with more stability (DTX, PAX and DOX drugs), the Coarse Grained simulations were repeated over a longer period of time (1100 ns) and at the best condition of nanocarrier concentration (concentration of 20% of PLGA-PEG-Rf polymer). The results, which are quite similar to the All atom simulations of these drugs, are shown in Fig. 7.

## Experimental

### Zeta

Potential at the slipping/shear plane of a colloid particle moving under electric field, is Zeta potential (ZP), also known as electrokinetic potential which is used to measure colloid stability. Volume of work that needs to be done to bring a unit positive charge from infinity to the surface without any acceleration, is surface electric potential. Zeta potential ranges are 10 to 20 mV (which is relatively stable), to 30 mV (which is moderately stable) and > ± 30 mV (which is highly stable).

**Figure 8:**
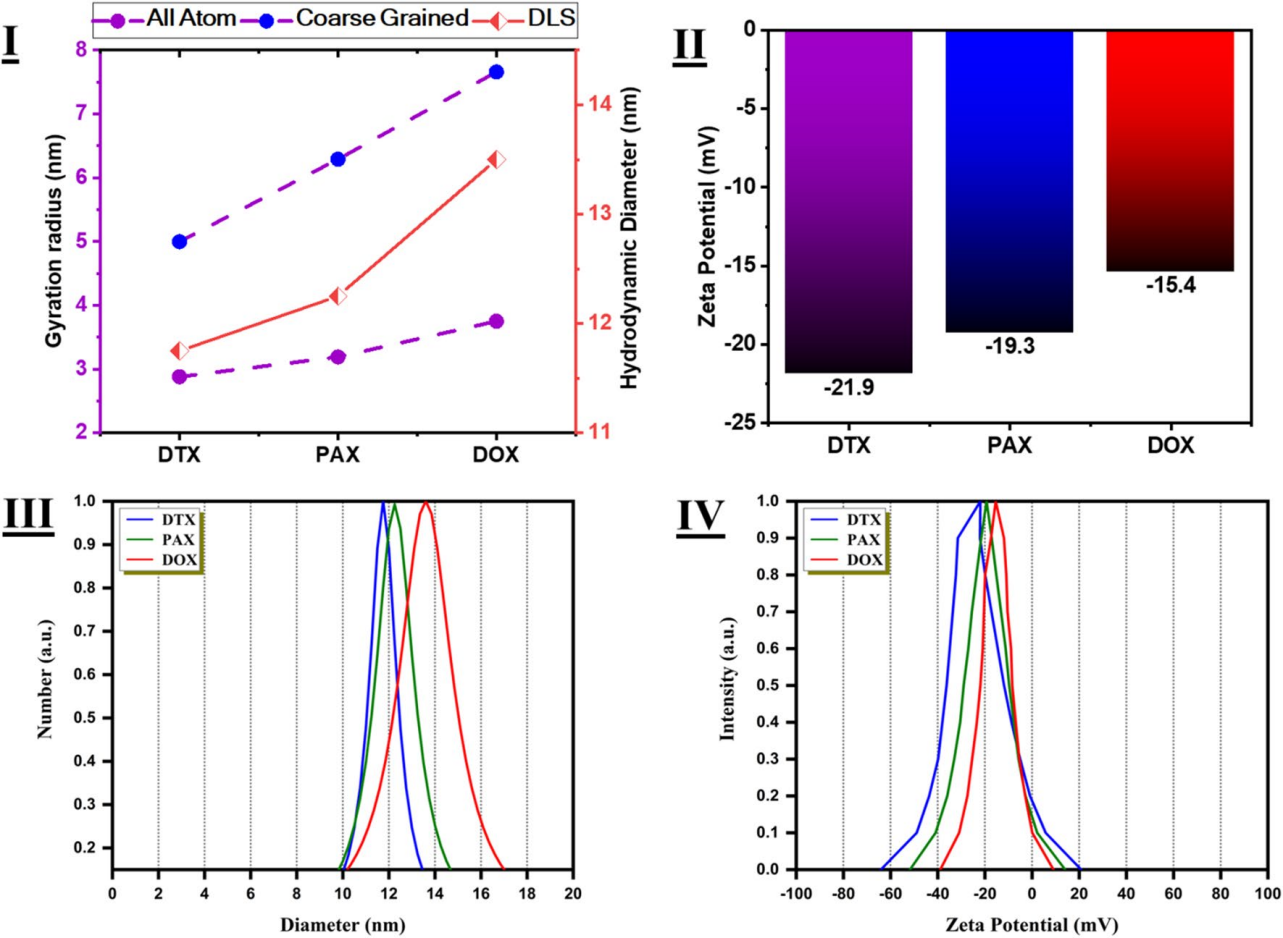
DLS testes and comparing with Simulation data for the 3 best drugs at the best concentration. (**I**) Comparing Hydrodynamic diameter and final value of Gyration radius at All Atom and Coarse Grained simulations (**II**) Zeta Potential of experimental data for the 3 best drugs at the best concentration. (**III**) and (**IV**) DLS and Zeta Potential tests for 3 best drugs at the best concentration.

Van der Waals force is dependent on Hamaker constant which corresponds to the difference between dispersant and refractive index (RI) of the particle. Thus, if Hamaker constant is low, so that van der Waals forces become low and then 10 to 15 mV (electrostatic repulsion reflected by low ZP) could be enough to name a colloid “stable”. Figure 8 displays Zeta potential of the Drug samples. Zeta-Potential results of DTX sample with − 21.9 mV showed moderately stability. Also, PAX and DOX samples with − 19.3 mV, and − 15.4 mV showed relatively stability.

### DLS

Generally public size threshold of 1 to 100 nm will be assumed as nanomaterials though still nanoscale materials should have at least one dimension ≤ 100 nm. Dynamic light scattering (DLS)—also known as quasi-elastic light scattering or photon correlation spectroscopy have arisen as quiet top method to study hydrodynamic size of nanoparticles. For DLS measurements, samples should be homogeneous. According to the number curves, average particle size of samples is less than 50 nm. Figure 8 displays DLS of the Drug samples. DTX, PAX, and DOX samples with ~ 11.75, ~ 12.25, and ~ 13.50 average nanometer diameters were shown in number curve plot.

## Discussion

As previously mentioned, R_g_ is an important factor in the evaluation of particle stability and represents the root-mean-square distance of each nanoparticle to its center of mass. R_g_ is the radius of accumulation resulting from the accumulation of molecules. The stronger attraction energy between the molecules causes the molecules to become closer to each other. The higher the energy of attraction between molecules, the lower the chance of molecular aggregation due to external forces. Thus, the gyration radius can represent the energy intensity between the molecules and the stability of the system. In this regard, Rg is calculated for all systems in the presence of drug molecules, which are entrapped within polymer chains.

Assessment of the Gibbs free energy showed that NPs based on _PLGA 3 kDa-PEG2 kDa_ have a lower level of energy (i.e., better stability). We can attribute the detected difference to the molecular structure of the repeating units of both polymer strands. In other words, having more lactide in the composition leads to a higher content of the methyl group in the chain structure. It results in the loss of flexibility that prevents molecules from attracting each other due to steric hinderance^43^. The obtained results from simulations are in line with previous reports, in which the ratio of lactic:glycolic acids tunes the performance of the polymer in vivo and in vitro^44,45^. It clearly shows that a balance between hydrophobic and hydrophilic blocks in the polymers appears to be effective in the stability of moiety-conjugated particles^46^. Gibbs free energy assessment of the results with incorporated drug molecules revealed that stable samples based on both polymers (PLGA_3kDa_-PEG_2kDa_ and PLGA_4.5 kDa_-PEG_2kDa_) load more drug molecules in compared with the other counterparts. In line with previous studies, the vdW interaction is the most dominant interaction in the formation of NPs and drug loadings. From the energy calculation point of view, the addition of drug molecules to the NPs improved their stability. However, stability occurs at the same ratio of ligand-attached polymer in the composition, which was previously detected without the presence of DTXs. The control samples (at PP:PPR 10:0) had larger Rg, higher energy levels, and lower drug loading efficiency, which indicates that riboflavin on the surface of NPs improves the interaction between drugs and NPs and stimulates more drug content in the particles.

The drug’s binding affinity to a particular biopolymer depends mainly on its hydrophobicity, physical interactions, and molecular features^47^. Our observations of the drug loading efficiency can be tied to the hydrophobic interactions of PLGA, RF, and DTX. The ligand and drugs are water-insoluble and hydrophobic. The more hydrophobic ligands there are in the composition, the more drugs there are in the content of the final nanoparticle. It should be mentioned that the moiety and RF-conjugated polymer chains are located on the surface of NPs. As noted previously, at the ends of polymer chains, RF molecules bend toward the particle mass center, but neighboring PEG chains prevent them from being completely confined to the mass. It is a valuable finding that all the RF molecules used in the preparation of targeted particles are available to be used in the targeting cells. Concludingly, NPs consist of a hydrophobic core of PLGA and entrapped DTX with a hydrophilic PEG shell and RF ligand over the surface.

The present work explains a computer simulation method to evaluate the ability of polymeric nanoparticles to incorporate poorly water-soluble antitumor agents without further need for time-consuming and expensive experiments. In this paper, we have reported a molecular dynamics simulation approach to estimate the drug entrapment efficiency of riboflavin-conjugated PLGA-based NPs with a PEG shell as well as the effect of the moiety on the stability of particles. To the best of our knowledge, we report for the first time the NP drug loading content with respect to the optimization of targeting ligand density.

## Conclusion

From a bird’s eye view, we conclude that Rg can be used as a meaningful scale of stability without the need for further costly computations. Similar trends in the Gibbs free energy diagrams and Rg plots were observed, as the minimum of both occurred at the same compositions. Of course, we also used experimental tests to confirm the results of the simulation. We observed from the current report and our previous paper^24^ that further experimental and computational analysis with PLGA polymers with an equal content of lactic and glycolic acid provides satisfactory results. Therefore, in future papers, we will use polymers with equal ratios of hydrophilic and hydrophobic segments.

## Supplementary Information

Supplementary Information.
