# Supplementary material for: Biomolecular engineering of drugs loading in Riboflavin-targeted polymeric devices: simulation and experimental
Source: Sci Rep. 2022 Mar 24;12:5119. doi: 10.1038/s41598-022-09164-2 (PMC8948184; doi:10.1038/s41598-022-09164-2)
Supplement: Supplementary file 1 — Supplementary Information. [file 41598_2022_9164_MOESM1_ESM.docx]

**Biomolecular Engineering of Drugs Loading in Riboflavin Targeted Polymeric Devices : Simulation and Experimental**

Mohammad Khedri ^a^, Mostafa keshavarz Moraveji ^a,^*

^a^ Department of Chemical Engineering, Amirkabir University of Technology (Tehran Polytechnic), No. 350, Hafez Ave, Valiasr Square, 15916-34311, Tehran, Iran

*Corresponding Author: [*moraveji@aut.ac.ir*](mailto:moraveji@aut.ac.ir)


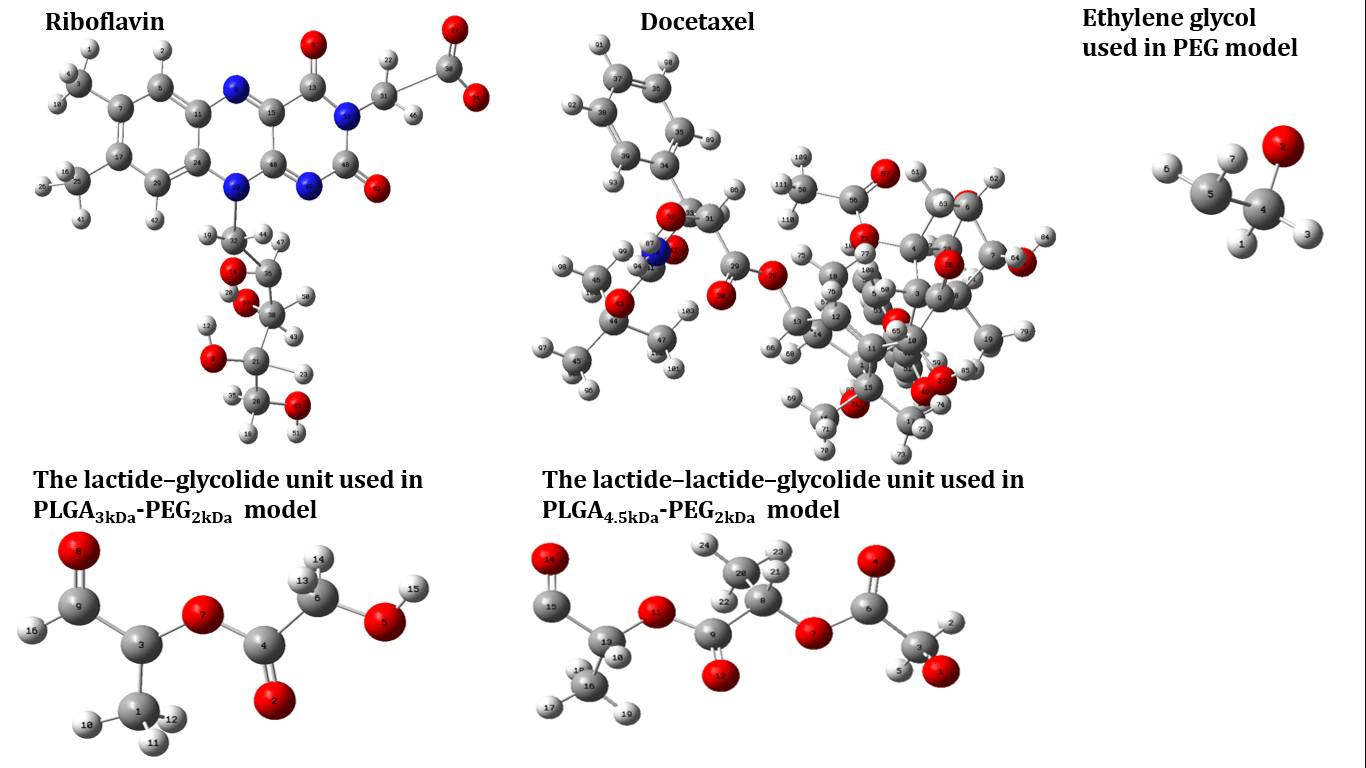


***Figure S1. Atom types of molecules and polymers used in simulations.*** *To mimic the real conditions, the monomer of two PLGA-PEG polymers has a different number of lactide-glycolide units. Details on the type and charge of atoms according to the numbers are provided in Table S1.*

Table S1 provides further detail about the molecule structures used in the simulations.

| **Table S1**. Specification of the OPLS-AA atom types and partial charges used in the simulations. | | | | | | | | | | | |
| --- | --- | --- | --- | --- | --- | --- | --- | --- | --- | --- | --- |
| **Docetaxel** | | | | | | | **Riboflavin** | | **The monomer used in PLGA_3kDa_-PEG_2kDa_** | | |
| NO. | Atom | Charge | NO. | Atom | Charge | NO. | Atom | Charge | NO. | Atom | Charge |
| 1 | C00 | 0.2091 | 57 | O1M | -0.4263 | 1 | C00 | -0.0041 | 1 | H00 | 0.3444 |
| 2 | C01 | 0.1265 | 58 | C1N | -0.3801 | 2 | C01 | 0.1634 | 2 | O01 | -0.5099 |
| 3 | C02 | -0.1273 | 59 | H1O | 0.1365 | 3 | C02 | -0.3509 | 3 | C02 | -0.0017 |
| 4 | C03 | 0.1281 | 60 | H1P | 0.1356 | 4 | O03 | -0.4288 | 4 | C03 | 0.3645 |
| 5 | C04 | 0.0079 | 61 | H1Q | 0.1529 | 5 | N04 | -0.571 | 5 | H04 | 0.0785 |
| 6 | C05 | -0.2041 | 62 | H1R | 0.1191 | 6 | C05 | 0.488 | 6 | H05 | 0.0785 |
| 7 | C06 | 0.2192 | 63 | H1S | 0.1191 | 7 | C06 | 0.5732 | 7 | O06 | -0.2167 |
| 8 | C07 | -0.1489 | 64 | H1T | 0.0829 | 8 | N07 | -0.652 | 8 | O07 | -0.4362 |
| 9 | C08 | 0.3891 | 65 | H1U | 0.0982 | 9 | O08 | -0.3554 | 9 | C08 | -0.4679 |
| 10 | C09 | 0.1579 | 66 | H1V | 0.1684 | 10 | C09 | 0.5054 | 10 | C09 | -0.0784 |
| 11 | C0A | -0.1474 | 67 | H1W | 0.1248 | 11 | C0A | -0.0361 | 11 | C0A | 0.2589 |
| 12 | C0B | -0.1162 | 68 | H1X | 0.1248 | 12 | O0B | -0.3565 | 12 | H0B | 0.0314 |
| 13 | C0C | 0.0433 | 69 | H1Y | 0.1137 | 13 | N0C | -0.1318 | 13 | H0C | 0.0314 |
| 14 | C0D | -0.2467 | 70 | H1Z | 0.1137 | 14 | C0D | -0.0472 | 14 | H0D | 0.0314 |
| 15 | C0E | -0.0144 | 71 | H20 | 0.1137 | 15 | C0E | 0.1598 | 15 | O0E | -0.545 |
| 16 | C0F | -0.2873 | 72 | H21 | 0.0967 | 16 | N0F | -0.5311 | 16 | H0F | 0.037 |
| 17 | C0G | -0.2307 | 73 | H22 | 0.0967 | 17 | C0G | -0.0347 | **The monomer used in PLGA_4.5kDa_-PEG_2kDa_** | | |
| 18 | C0H | -0.1985 | 74 | H23 | 0.0967 | 18 | C0H | -0.1253 | 1 | O00 | -0.516 |
| 19 | C0I | -0.2484 | 75 | H24 | 0.1021 | 19 | C0I | -0.01 | 2 | C01 | -0.134 |
| 20 | C0J | -0.0038 | 76 | H25 | 0.1021 | 20 | C0J | -0.2623 | 3 | C02 | -0.049 |
| 21 | O0K | -0.6445 | 77 | H26 | 0.1021 | 21 | C0K | -0.17 | 4 | C03 | -0.187 |
| 22 | O0M | -0.4152 | 78 | H27 | 0.1131 | 22 | C0M | -0.1595 | 5 | O04 | -0.312 |
| 23 | O0N | -0.3462 | 79 | H28 | 0.1131 | 23 | C0N | -0.0298 | 6 | H05 | 0.08 |
| 24 | O0O | -0.3435 | 80 | H29 | 0.1131 | 24 | C0O | 0.1619 | 7 | H06 | 0.0497 |
| 25 | O0P | -0.6819 | 81 | H2A | 0.1027 | 25 | C0P | -0.2278 | 8 | H07 | 0.0497 |
| 26 | O0Q | -0.3393 | 82 | H2B | 0.1027 | 26 | C0Q | -0.191 | 9 | H08 | 0.0497 |
| 27 | O0R | -0.7299 | 83 | H2C | 0.4054 | 27 | C0R | -0.0805 | 10 | C09 | 0.353 |
| 28 | O0S | -0.3227 | 84 | H2D | 0.4343 | 28 | O0S | -0.5784 | 11 | C0A | 0.0418 |
| 29 | C0T | 0.4513 | 85 | H2E | 0.4407 | 29 | O0T | -0.3829 | 12 | O0B | -0.429 |
| 30 | O0U | -0.3995 | 86 | H2F | 0.1516 | 30 | O0U | -0.4283 | 13 | C0C | -0.213 |
| 31 | C0V | 0.0952 | 87 | H2G | 0.4104 | 31 | O0V | -0.4579 | 14 | O0D | -0.297 |
| 32 | O0W | -0.6401 | 88 | H2H | 0.1818 | 32 | H0W | -0.0227 | 15 | H0E | 0.1128 |
| 33 | C0X | 0.2536 | 89 | H2I | 0.1469 | 33 | H0X | -0.0227 | 16 | H0F | 0.0741 |
| 34 | C0Y | -0.1301 | 90 | H2J | 0.1344 | 34 | H0Y | 0.1532 | 17 | H0G | 0.0741 |
| 35 | C0Z | -0.1338 | 91 | H2K | 0.1452 | 35 | H0Z | 0.1532 | 18 | H0H | 0.0741 |
| 36 | C10 | -0.1369 | 92 | H2M | 0.1425 | 36 | H10 | 0.1523 | 19 | C0I | 0.3218 |
| 37 | C11 | -0.1203 | 93 | H2N | 0.1384 | 37 | H11 | 0.1667 | 20 | C0J | 0.2073 |
| 38 | C12 | -0.1272 | 94 | H2O | 0.5577 | 38 | H12 | 0.0865 | 21 | O0K | -0.502 |
| 39 | C13 | -0.129 | 95 | H2P | 0.1168 | 39 | H13 | 0.0865 | 22 | H0M | -0.051 |
| 40 | N14 | -1.1228 | 96 | H2Q | 0.1168 | 40 | H14 | 0.0865 | 23 | H0N | -0.051 |
| 41 | C15 | 0.7437 | 97 | H2R | 0.1168 | 41 | H15 | 0.0799 | 24 | O0O | -0.749 |
| 42 | O16 | -0.5549 | 98 | H2S | 0.1184 | 42 | H16 | 0.0799 | **Ethylene glycol** | | |
| 43 | O17 | -0.3507 | 99 | H2T | 0.1184 | 43 | H17 | 0.0799 | 1 | H00 | 0.12 |
| 44 | C18 | 0.2082 | 100 | H2U | 0.1184 | 44 | H18 | 0.1448 | 2 | C01 | 0.0822 |
| 45 | C19 | -0.2812 | 101 | H2V | 0.1171 | 45 | H19 | 0.1448 | 3 | C02 | 0.0081 |
| 46 | C1A | -0.2803 | 102 | H2W | 0.1171 | 46 | H1A | 0.1194 | 4 | H03 | 0.12 |
| 47 | C1B | -0.3048 | 103 | H2X | 0.1171 | 47 | H1B | 0.0556 | 5 | O04 | -0.5065 |
| 48 | C1C | 0.523 | 104 | H2Y | 0.2012 | 48 | H1C | 0.0556 | 6 | H05 | 0.0881 |
| 49 | O1D | -0.3993 | 105 | H2Z | 0.1094 | 49 | H1D | 0.3246 | 7 | H06 | 0.0881 |
| 50 | C1E | -0.1468 | 106 | H30 | 0.1363 | 50 | H1E | 0.3779 |  |  |  |
| 51 | C1F | -0.0539 | 107 | H31 | 0.1617 | 51 | H1F | 0.0325 |  |  |  |
| 52 | C1G | -0.1824 | 108 | H32 | 0.1589 | 52 | H1G | 0.4177 |  |  |  |
| 53 | C1H | -0.101 | 109 | H33 | 0.1671 | 53 | H1H | 0.3927 |  |  |  |
| 54 | C1I | -0.1519 | 110 | H34 | 0.1671 | 54 | H1I | 0.4068 |  |  |  |
| 55 | C1J | -0.0716 | 111 | H35 | 0.1671 |  |  |  |  |  |  |
| 56 | C1K | 0.5059 |  |  |  |  |  |  |  |  |  |


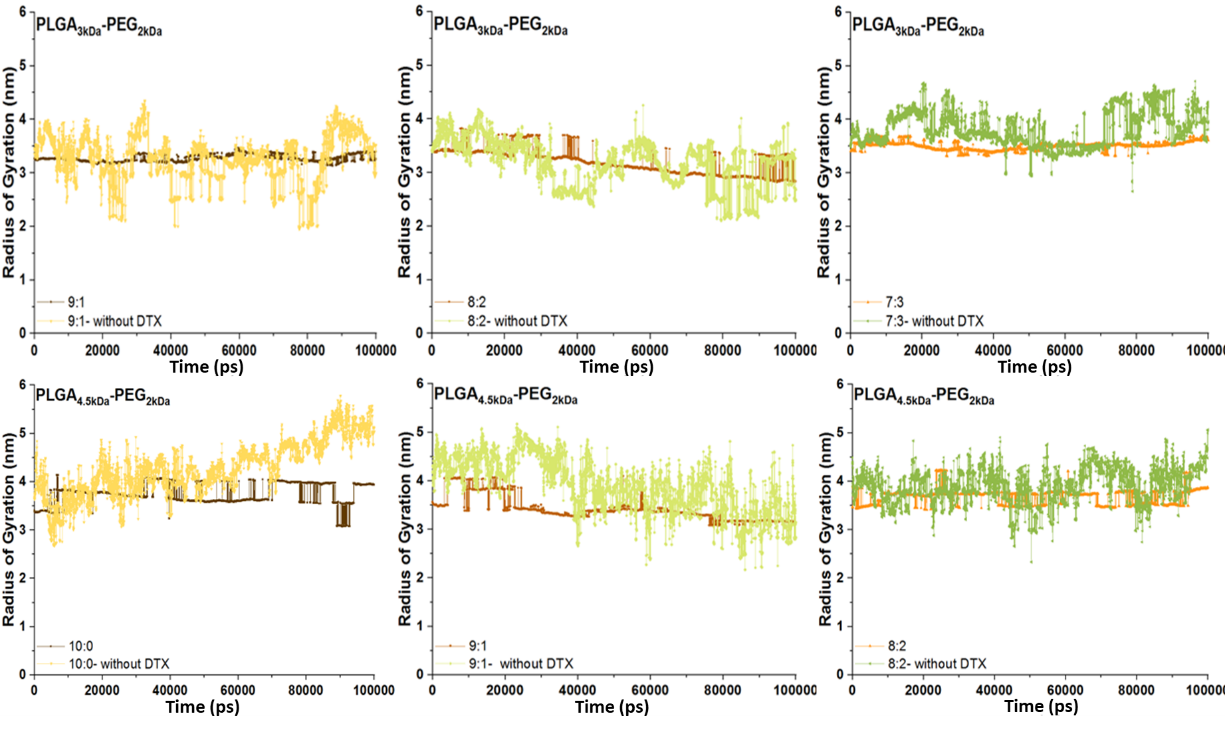


***Figure S2. Fluctuation of Rg for NPs formation process.*** *At each PP:PPR drug-loaded and unloaded NPs are compared which declares the lower oscillations and as a result better stability of the DTX containing particles.*


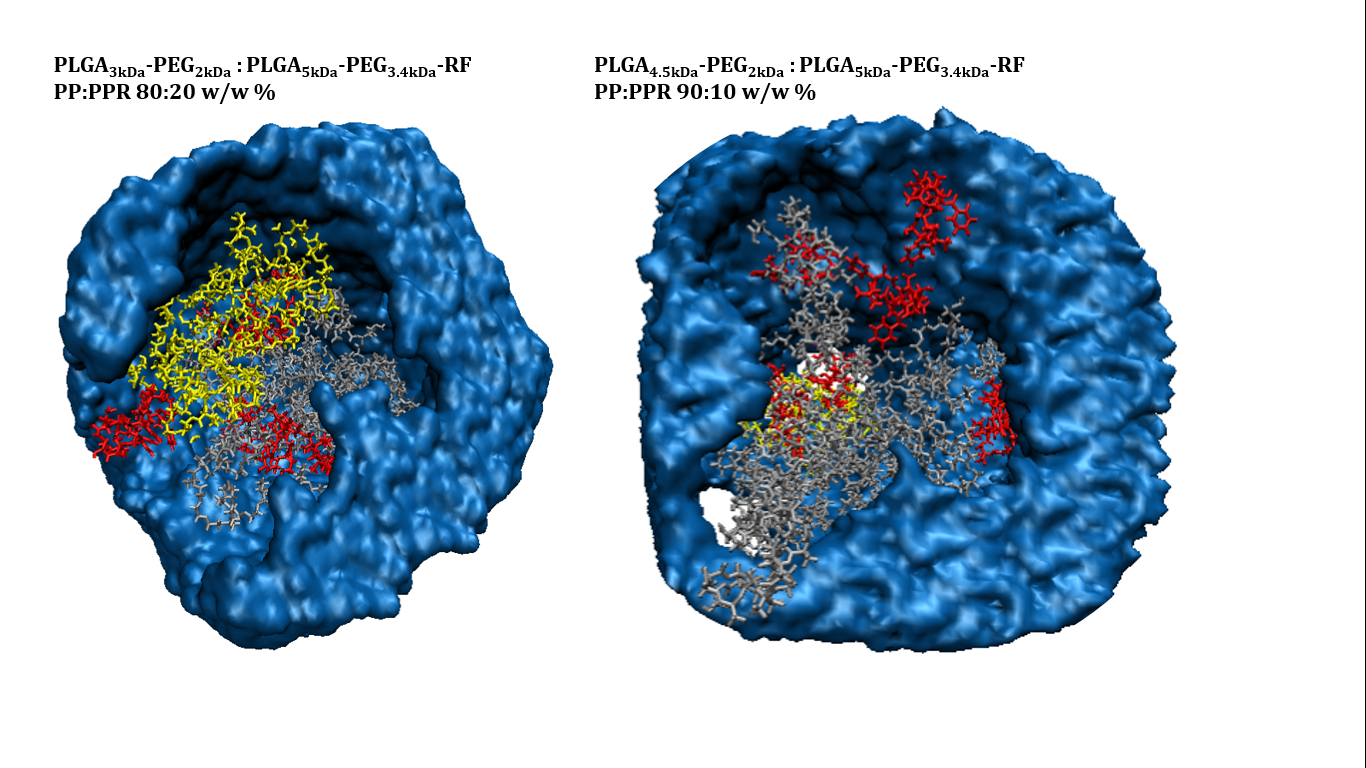


***Figure S3. Water molecules around NPs.*** *Left: optimized sample (8:2) for PLGA_3kDa_-PEG_2kDa_ shows that water molecules surrounded polymer chains containing DTX molecules. Right: optimized sample (9:1) for PLGA_4.5kDa_-PEG_2kDa_ is hydrophobic and that water molecules surrounded polymer chains containing antitumor drugs. Results are in good agreement with previous result.*


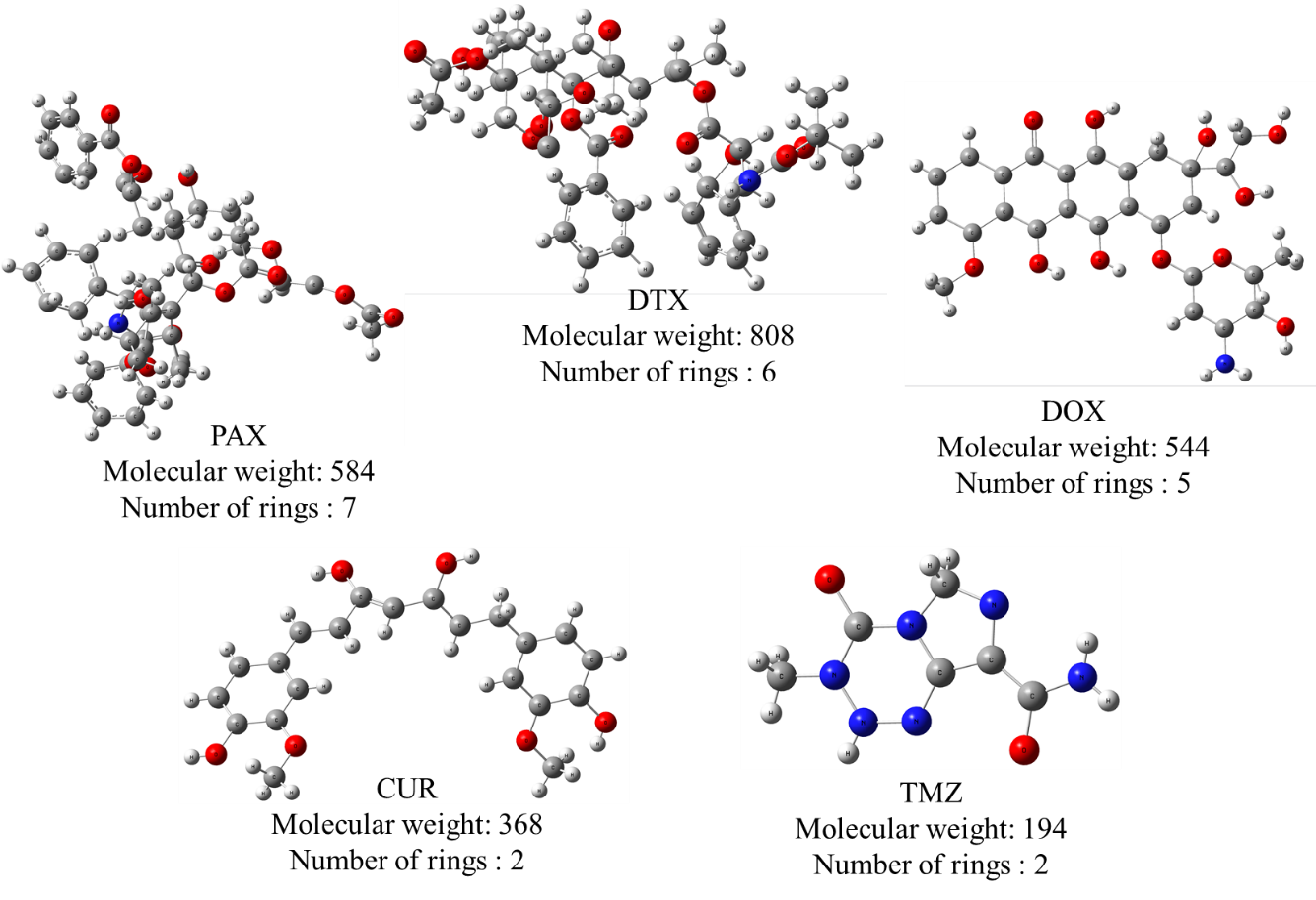


***Figure S4. Molecular weight and Number of rings and Structures of DTX, PAX, DOX, CUR and TMZ drugs.*** *(*Nitrogen in the structure is blue, Carbon is silver, Oxygen ins red and Hydrogen is white.)
